# Exercise and calorie information on menus is not enough to improve food choices in Hispanic adults

**DOI:** 10.1186/1550-2783-12-S1-P3

**Published:** 2015-09-21

**Authors:** Brooke Bouza, Jessica Fellow, Maxine Lorenz, Lauren Rutledge, Manall Jaffery, Beverley Adams-Huet, Lyn Dart, Phil Esposito, Meena Shah

**Affiliations:** 1 Department of Kinesiology, Texas Christian University, Fort Worth, TX 76129, USA; 2 Department of Clinical Sciences, UT Southwestern Medical Center at Dallas, Dallas, TX 75390, USA; 3 Department of Nutritional Sciences, Texas Christian University, Fort Worth, TX 76129, USA

## Background

Hispanics are a fast growing population in the U.S. with a high prevalence of obesity or overweight. Eating out frequently in restaurants is linked with weight gain, and several strategies to improve food choices from menus have been studied. Some of the strategies that may be effective include displaying the amount of exercise needed to burn the food calories, rank ordering the food items by calorie content, and showing both calorie content of foods and the recommended calories together. However, most of the participants in the previous studies were non-Hispanic. Hispanics engage in sports activities and eat out in restaurants just like non-Hispanics and whether the exercise and calorie labels will affect their food choices needs to be determined.

## Methods

Three-hundred and seventy-two Hispanic adults (18-65 years) were randomized to a menu with no labels (NL) (n = 127), a menu with rank ordered calorie labels plus a statement on the number of calories recommended per meal (CL) (n = 123), or a menu with rank ordered exercise labels showing the duration of brisk walking necessary to burn the food calories (EL) (n = 122). Food and drink choices were identical on each menu. Participants were given the assigned menu and instructed to circle the food and drink items they would order, as if having lunch in a fast-food restaurant. The menus were developed in both English and Spanish and the participants decided which language version they preferred. Participants were not informed about the study purpose and were paid $10 for completing the study. The effects of menu condition on the number of calories and percent energy from fat ordered were assessed by analysis of variance.

## Results

There were no differences in calories ordered (mean ± standard deviation: NL: 792 ± 378 kcal; CL: 829 ± 415 kcal; EL: 867 ± 528 kcal; p = 0.41) or percent energy from fat ordered (NL: 35.0 ± 7.3%; CL: 34.4 ± 8.3%; EL: 35.4 ± 8.2%; p = 0.64) by menu condition. Adjustment for age, weight status, gender, hunger level, price, education, and whether or not they used the English or Spanish version of the menus did not affect the number of calories ordered.

## Conclusions

The EL and CL menus did not affect the number of calories or percent energy from fat ordered by Hispanic adults. This study indicates that it is not enough to just provide information on menus. Coaches, fitness trainers, and nutritionists also need to educate their Hispanic clients about the importance of controlling calorie intake when eating out.

